# The clinically licensed antifungal drug itraconazole inhibits influenza virus *in vitro* and *in vivo*

**DOI:** 10.1080/22221751.2018.1559709

**Published:** 2019-01-16

**Authors:** Sebastian Schloer, Jonas Goretzko, Alexander Kühnl, Linda Brunotte, Stephan Ludwig, Ursula Rescher

**Affiliations:** a Institute of Medical Biochemistry, Centre for Molecular Biology of Inflammation, University of Muenster, Muenster, Germany; b Interdisciplinary Centre for Clinical Research, University of Muenster, Muenster, Germany; c Cluster of Excellence “Cells in Motion”, University of Muenster, Muenster, Germany; d Institute of Virology, Center for Molecular Biology of Inflammation, University of Muenster, Muenster, Germany

**Keywords:** Influenza A virus, posaconazole, itraconazole, interferon response, cellular cholesterol, host cell factors, mouse model, drug repurposing

## Abstract

Influenza A virus (IAV) is a common pathogen of respiratory disease. The IAV-induced seasonal epidemics and the sporadic pandemics are associated with high morbidity and mortality. Therefore, effective protection and therapy for IAV infections is an important challenge in countering this public health threat. Because vaccinations only protect against known circulating strains, and the currently available antivirals pose the risk of resistance formation, drugs targeting host cell factors needed for viral replication offer a promising therapeutic approach. In this study, we describe the use of the antifungal therapeutics posaconazole and itraconazole in the therapy of IAV. We show that both drugs efficiently inhibit the propagation of IAV in the cell culture model without being cytotoxic. The mode of action is probably based on several targets and includes both a priming of the interferon response and the induced imbalance of cellular cholesterol. The antiviral effect of itraconazole could be confirmed in the mouse model, where the administration of itraconazole led to a drastic reduction in mortality and a significant increase in the survival rate. Thus, our data indicate a promising therapeutic potential of at least itraconazole in influenza therapy.

## Introduction

IAV causes seasonal epidemics affecting millions of people each year and poses an enormous threat to public health. Thus, efficient protection and therapy for IAV infection is an important challenge for global healthcare. Vaccination is the main preventive measure, but it is limited to the known circulating strains, as the vaccines do not protect against outbreaks caused by antigenically divergent strains [1–4]. The currently licensed antivirals are not very effective and pose the risk of viral resistance. For this reason, new strategies and approaches are needed that provide broad protection against IAV. One such approach is the development of drugs that target host cell factors required for viral replication [5,6].

The initial steps during the virus life cycle are attachment to sialic acid on the surface of host cells, followed by exploitation of cellular uptake mechanisms [7]. Many enveloped viruses, including IAV and VSV, are entering the cells by trafficking through early to late endosomes, where the pH-dependent fusion of viral and endosomal membrane occurs, thus allowing the release of the viral genome into the cytoplasm of the host cell [8]. In the case of IAV, the viral ribonucleoprotein complexes (vRNPs) are then translocated to the nucleus, where viral replication and transcription takes place [9]. Therefore, the virus entry process is a crucial limiting step in the virus infection cycle and may thus be a promising target for antiviral intervention.

A solid body of evidence links the cellular cholesterol metabolism with the innate immune response [10–15]. Notably, type I interferon (IFN) signalling decreases cholesterol synthesis and elevates cholesterol import in macrophages [16]. In return, limiting cholesterol biosynthesis enhances type I IFN expression and stimulates the expression of IFN-dependent genes. Both observations are regarded as critical events of the antiviral response [17]. The activation of the IFN-induced immune response triggers the expression of several antiviral proteins like Interferon Induced Transmembrane Protein 3 (IFITM3) that interfere with the fusion of viral and endosomal membrane [6]. In cells transfected with IFITM3, an endosomal accumulation of cholesterol has been observed, which is reported to function in the IFITM3 antiviral activity [18,19].

Because our previous work indicated a crucial role for endosomal cholesterol balance in the release and infectivity of IAV progeny, we investigated the antiviral capability of two well-studied, systemic triazole derivate drugs itraconazole and posaconazole [10,20]. These drugs display antifungal activity due to the inhibition of cytochrome P450 (CYP) enzymes, particularly the lanosterol 14α-demethylase, which is essential for the ergosterol biosynthesis in fungi, thus inhibiting fungal growth and membrane function [21]. In mammalian cells, however, its inhibition impairs homeostasis and *de-novo* synthesis of cholesterol [22]. Furthermore, both anti-fungal compounds inhibit the late endosomal/lysosomal (LE/L) cholesterol export by blocking the cholesterol-transferring membrane protein Niemann-Pick C1 (NPC1), resulting in accumulation of cholesterol in LE/L [23]. Here, we explore the antiviral capacity of both antifungals for the treatment of infections caused by various IAV and IBV subtypes *in vitro* and *in vivo*.

## Results

### Itraconazole and posaconazole inhibit the propagation of IAV and VSV

**Figure 1.**
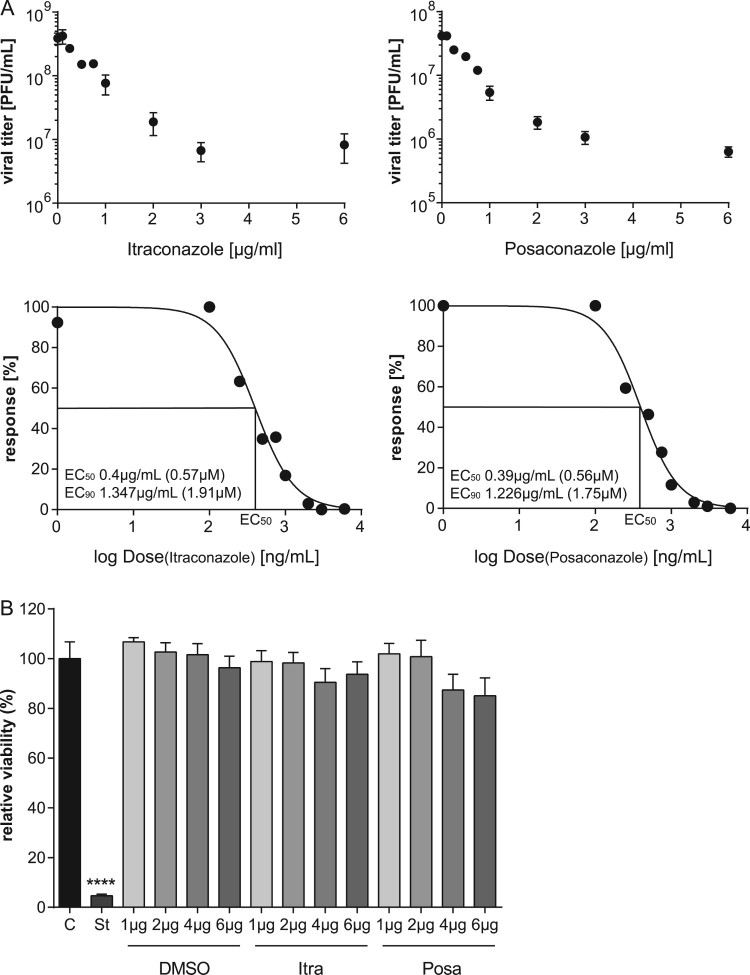
The antifungals itraconazole and posaconazole block IAV infection at non-cytotoxic concentrations. (A) Dose–response curve of itraconazole and posaconazole in A549 cells. Cells were treated with either DMSO, posaconazole (Posa) or itraconazole (Itra) for 16 h, and subsequently infected with IAV strain PR8M (MOI = 0.1). Virus titers were converted to percentages, posaconazole and itraconazole concentrations were log-transformed, and EC_50_ and EC_90_ values were calculated from the semi-logarithmic fitted curves. Data represent mean viral titers ± SEM of three independent experiments. (B) MTT assay of A549 cells treated with either the protein kinase inhibitor staurosporine (St), the solvent DMSO, itraconazole (Itra) or posaconazole (Posa) at the indicated concentrations for 24 h. Data represent means ± SEM, of eight independent experiments; one-way ANOVA with Dunnett’s multiple comparison tests, ****p ≤ 0.0001.

**Figure 2.**
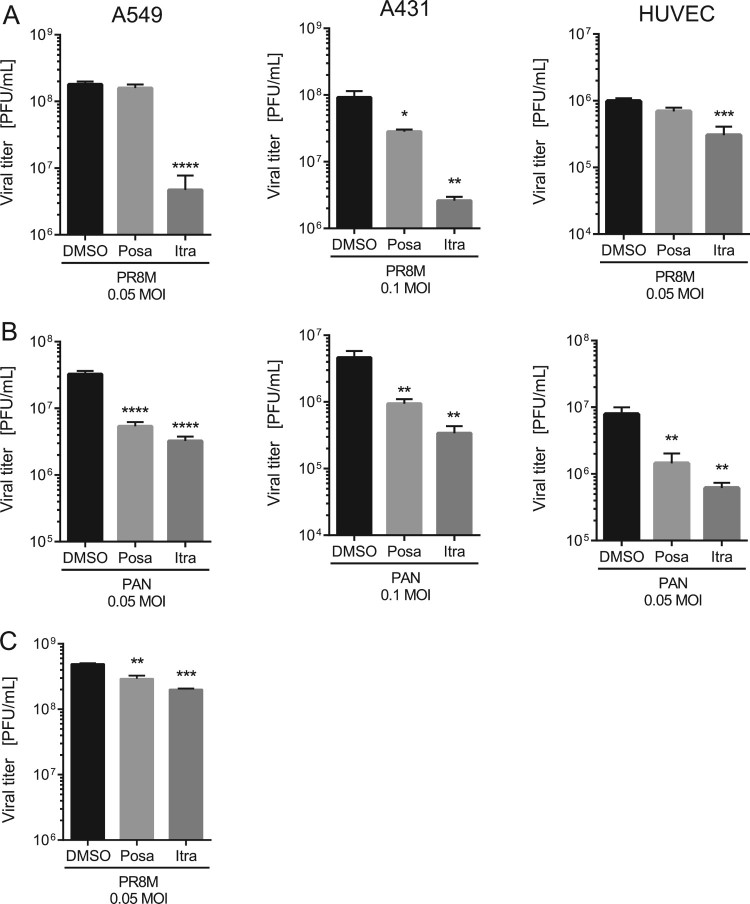
The antiviral activities are not subtype or cell line specific. A549, A431 cells or HUVECs were treated with either DMSO, posaconazole (Posa) or itraconazole (Itra) for 16 h, and subsequently infected with the indicated MOI of (A) IAV strain PR8M or (B) PAN for 24 h. (C) Postinfection treatment (2 h p.i.) of PR8M-infected A549 cells with either control, Itra or Posa. Data represent mean viral titers ± SEM, *n* = 5; one-way ANOVA with Dunnett’s multiple comparison tests, ****p ≤ 0.0001;***p ≤ 0.001; **p ≤ 0.01; *p ≤ 0.05.

To evaluate a potential antiviral activity of the antifungals itraconazole and posaconazole, we measured the relationship between drug dose and infection outcome after IAV infection in the human lung epithelial cell line A549, a cell line commonly used for IAV propagation. Both antifungals are NPC1 inhibitors [23] and since we identified in our previous work a 16 h period of NPC1 blockade as a robust antiviral barrier to IAV infections [10,24], this time period was also chosen for drug pretreatment. As shown in Figure 1(A), both drugs efficiently inhibited the replication of the H1N1 strain PR8M. The 50% effective concentrations (EC_50_ values) were seen at low micromolar concentrations, and higher doses reduced IAV propagation up to 98%. To verify that the reduction in viral replication was due to an inhibition of the IAV replication cycle and not to an indirect effect on the host cell, we next evaluated the potential cytotoxic effects of both antifungals. We performed an MTT-assay which measures the activity of mitochondrial dehydrogenases [25], that is only active in proliferating viable cells. Cell viability was examined in cells which had been exposed for 24 h to solvent DMSO, or increasing concentrations of itraconazole and posaconazole. As shown in Figure 1(B), staurosporine, a potent and non-selective protein kinase inhibitor used as a positive control, caused massive cell death, while neither DMSO nor any of the itraconazole and posaconazole concentrations tested reduced the cellular viability in a significant manner, suggesting that the two antifungals actually inhibited IAV propagation. In order to explore the antiviral potential of itraconazole and posaconazole further, we infected A549 cells and the human skin epithelial cell line A431, which is also permissive for IAV [10], with various IAV subtypes (PR8M (H1N1), SC35M (H7N7), and PAN (H3N2)). Furthermore, the prototype rhabdovirus VSV was used as an additional enveloped virus. As expected, all viral infections yielded high virus titres 24 h p.i. in DMSO-treated control cells. Importantly, itraconazole pretreatment for 16 h significantly reduced viral titers for both IAV and VSV (Figure 2(A), B; Suppl. Fig. S1). In A549 and A431 cells pretreated with itraconazole, PR8M load 24 h p.i. was reduced up to 98% when cells were infected at 0.05 MOI. Reduced titers were also observed when cells were infected with higher viral doses (0.1 MOI), although the effect was less pronounced. Pretreatment with posaconazole was not as effective as itraconazole pretreatment, and both IAV and VSV infections were inhibited in A431 cells only (Figure 2(A,B); Suppl. Fig. S1). Because both the A549 as well as the A431 cell line are derived from cancer cells (human lung adenocarcinoma and epidermoid carcinoma, respectively), we investigated whether the antiviral capacity could be also observed in non-malignant primary human cells. We, therefore, used primary cells isolated from the umbilical cord vein (HUVEC), which can be efficiently infected with IAV [26]. As shown in Figure 2(A), propagation of the exemplary IAV strains PR8M was also successfully inhibited upon itraconazole treatment, whereas both drugs could block infection with the PAN strain (Figure 2(B)). Postinfection treatment (2 h p.i.) of PR8M-infected A549 cells resulted in reduced IAV titers compared to control-treated cells (Figure 2(C)), however, the antiviral activity was less prominent. Because in some seasons IBV can be the predominant type [27], we additionally explored the potential use of both antifungals for IBV infections. Interestingly, both substances also had a protective effect against IBV strain B/Lee/1940 (Suppl. Fig. S1).

### Itraconazole and posaconazole treatment primes the interferon response

**Figure 3.**
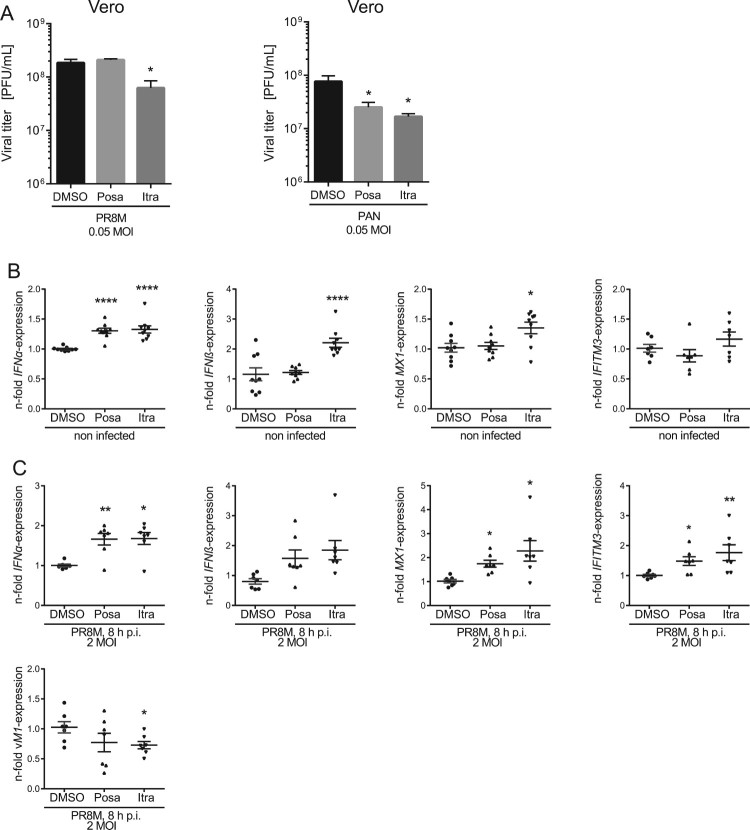
Itraconazole and posaconazole prime the IFN response. (A) PR8M and PAN virus titers upon posaconazole (Posa) and itraconazole (Itra) treatment of IFN-insensitive Vero cells. (B, C) qPCR analysis of the IFNs *IFNα* and *IFNβ* and the ISGs *IFITM3* and *MX1* in non-infected and (C) PR8M-infected A549 cells after 16 h treatment with either DMSO, itraconazole (Itra) or posaconazole (Posa). Samples were obtained from at least seven independent experiments and were run in triplicates. Expression levels of the genes of interest in the individual samples were normalized to GAPDH and ACTB. 2^−ΔΔCt^ was used to calculate the fold change of relative gene expression compared to control. Graphs show drug-induced fold difference in the respective genes relative to control in the individual samples, with the mean fold change superimposed. Note that in (B), all samples were uninfected, whereas in (C), all samples were IAV-infected. Statistical significance of the differences was evaluated by one-way ANOVA with Dunnett’s multiple comparison tests on ΔΔCt values. ****p ≤ 0.0001; ***p ≤ 0.001; **p ≤ 0.01; *p ≤ 0.05.

To determine whether the IFN response is involved in the antiviral capability of itraconazole and posaconazole, we employed Vero cells which are genetically defective in the production of IFN [28]. Of note, we still observed a decrease of viral titers upon infection with IAV or VSV, although less pronounced (Figure 3(A), Suppl Fig. S1). Viral load was reduced up to 50% in itraconazole-pretreated Vero cells when compared to the control cells, and only a small reduction in viral titers of VSV in posaconazole-pretreated Vero cells was detected. (Figure 3(A), Suppl Figure S1). Thus, our results point to a partial involvement of the IFN system in mediating an antiviral effect of itraconazole and posaconazole. Because both antifungals elicited a more prominent decrease in viral titers of enveloped viruses in A549 and A431 cells, we next asked whether there was an itraconazole-mediated effect on the IFN system. Type I IFNs induce the transcriptional upregulation of IFN-stimulated genes (ISGs), which are, therefore, widely used as surrogate markers for induction of the IFN response both in experimental and clinical approaches [29]. Expression of the ISG *MX1*, in particular, is exclusively induced via IFN, and is, therefore, a unique marker for IFN activity [29]. Thus, we performed qPCR analysis for the type I IFNs *IFNα* and *IFNβ* and the ISGs *MX1 and IFITM3.* Indeed, we found evidence for a weak induction of the IFN response. As shown in Figure 3(B), *IFNα* mRNA levels were moderately, albeit significantly elevated upon itraconazole and posaconazole treatment, whereas *IFNβ* levels were only altered upon itraconazole treatment. Notably, we confirmed that the enhanced basal expression level of *IFN*-α/β was accompanied by moderately increased *MX1*and *IFITM3* mRNA levels, suggesting weak alert of the cellular immune system prior to infection. Next, we explored the capacity of this weak induction observed in uninfected cells to affect a subsequent IAV infection. As shown in Figure 3(C), a more pronounced upregulation of *IFNα/β* and the ISGs was observed in drug-treated infected cells compared to control-treated infected cells, indicating a drug-induced priming.

### Itraconazole and posaconazole negatively affect IAV propagation within the first replication cycle

**Figure 4.**
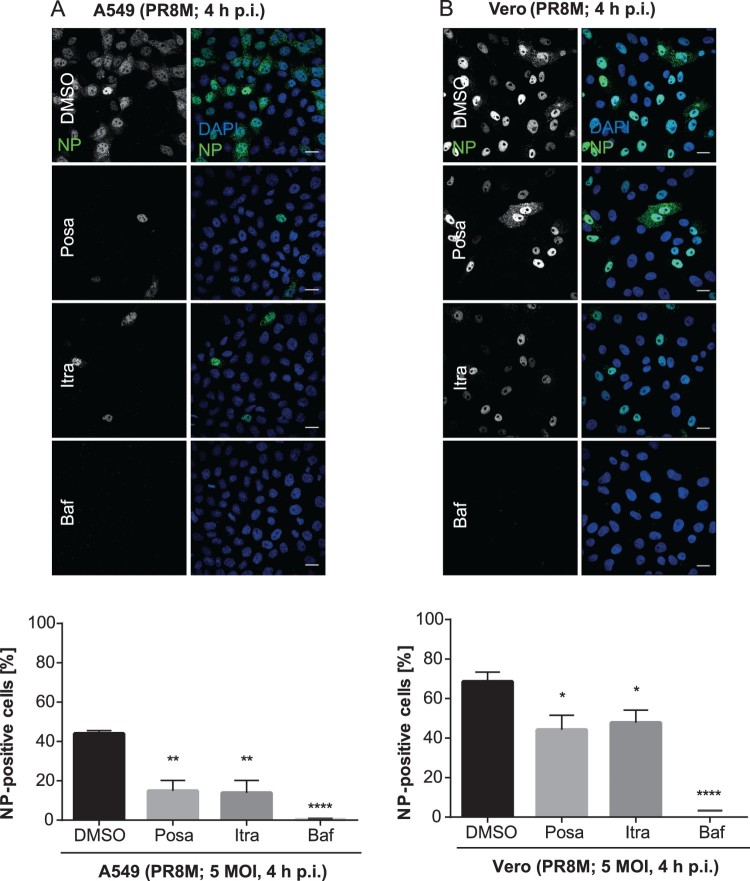
Itraconazole and posaconazole impair an early step in the IAV infection cycle. Cells pretreated with either the solvent DMSO, posaconazole (Posa), or itraconazole (Itra) for 16 h were infected with PR8M at the indicated MOI for 4 h. Bafilomycin A1 (Baf) served as a negative control. Nuclei were stained with DAPI, and infected cells were detected by nuclear vNP staining. Scale bar, 20 µm. To assess the percentages of successfully infected cells, the numbers of NP-positive nuclei were automatically quantified. Representative images are shown, and bar graphs represent the respective mean percentages ± SEM of NP-positive nuclei of >1000 infected cells. (A) A549 cells, three independent experiments, (B) Vero cells, Data represent means ± SEM *n* = 4. ****p ≤ 0.0001; ***p ≤ 0.001; **p ≤ 0.01; *p ≤ 0.05; one-way ANOVA followed by Dunnett’s multiple comparison test.

To examine if itraconazole and posaconazole affect an early step in IAV infection, we performed single-cycle infection assays. Therefore, A549 and Vero cells were treated for 16 h with the compounds and subsequently infected with PR8M for 4 h. The nuclear appearance of viral nucleoprotein (vNP) was quantified as an output of a successfully infected cell (Figure 4). In DMSO-treated control cells, 44% of A549 cell nuclei were NP-positive. Treatment of the host cells with Bafilomycin A1, an inhibitor of the vacuolar ATPase required for endosomal acidification, which efficiently blocks successful IAV/endosome fusion and release of the viral genome into the host cell cytoplasm, served as a control for efficient IAV inhibition, and a robust inhibition (less than 1% cells with NP-positive nuclei) was observed, as expected. Remarkably, a significantly lower percentage (14%) of cells were NP-positive when pretreated with either itraconazole or posaconazole (Figure 4(A)). In accordance with the data presented above, that the two drugs also have an antiviral effect in Vero cells, a significantly reduced number of NP-positive Vero cell nuclei was detected in the single-cycle assay (Figure 4(B)), suggesting that both drugs impaired IAV replication within the first cycle, independently of the IFN response.

**Figure 5.**
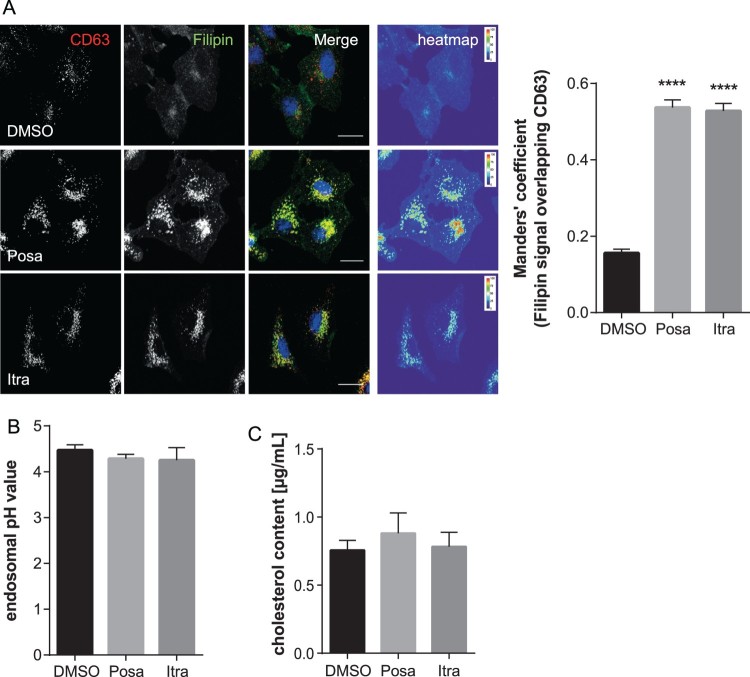
Itraconazole and posaconazole induce endolysosomal cholesterol storage without affecting endolysosomal acidification. (A) A549 cells treated for 16 h with either the solvent DMSO, posaconazole (Posa), or itraconazole (Itra) were stained for the LE/L marker protein CD63. Unesterified cellular cholesterol was visualized using filipin. DRAQ5 was used to label nuclei. Note that the digital images were pseudocolored. Scale bar, 20 µm. Colocalization coefficients of CD63 signals with filipin were quantitated from z-stacks. Bar graphs represent means ± SEM calculated from 20 individual cells per condition from two independent experiments. (B) A549 cells were treated as indicated for 16 h. Endosomal/lysosomal pH was measured by ratio imaging. pH values are means ± SEM of 30 cells for each condition from five independent experiments. (C) Cholesterol levels in cells treated with the solvent DMSO, posaconazole (Posa), or itraconazole (Itra). Data are expressed as mean cholesterol concentrations (in micrograms per millilitre) ± SEM from four independent experiments. ****p ≤ 0.0001; one-way ANOVA followed by Dunnett’s multiple comparison test.

Both itraconazole and posaconazole have been shown to inhibit cholesterol export from the endolysosomal (LE/L) compartment [23]. Interestingly, cholesterol imbalance within this compartment, where IAV endosomal escape occurs [30–32], is linked to impaired transfer of the viral genome into the host cell cytosol, thus reducing infection success [10,19,33]. We, therefore, used the LE/L marker protein CD63 to identify LE/L, and filipin to specifically label the free cellular cholesterol. As shown in Figure 5, cholesterol accumulated in LE/L of A549 cells upon treatment with both antifungal drugs. To verify and quantitate the endosomal cholesterol imbalance, we next determined the degree of overlap of the CD63 signals with the filipin signals using the Manders' coefficient. This well-established method quantifies the intensity correlation of two fluorophores, and revealed that LE/L cholesterol levels were indeed significantly increased in itraconazole- and posaconazole-treated cells (Figure 5(A)). Because the more acidic pH encountered in LE/L is a key factor for the fusion of the viral envelope with the endosomal membranes and the subsequent transfer of the viral genome into the host cell cytoplasm [30,34], we next assessed whether the accumulation of cholesterol in LE/L, induced by both compounds, is accompanied by aberrant endosomal pH. Thus, we performed a quantitative ratiometric fluorescence microscopy assay. As shown in Figure 5(B), both drugs did not affect the endosomal low pH (Figure 5(B) and Suppl. Fig. 3). In addition, we biochemically quantified and compared cholesterol levels of vehicle-treated and drug-treated cells. As shown in Figure 5(C), in both itraconazole- and posaconazole-treated cells, the cholesterol amounts were not significantly altered upon drug treatment, indicating that the more pronounced endosomal filipin signal was due to elevated levels of LE/L cholesterol (presumably via inhibition of the endosomal cholesterol transporter NPC1 [18]) rather than to a general change in cellular cholesterol.

**Figure 6.**
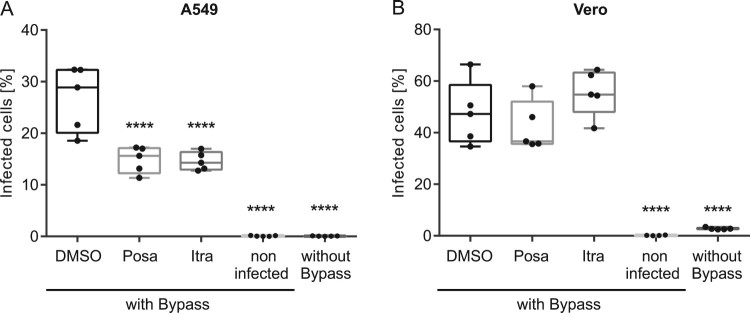
Effects of itraconazole and posaconazole on IAV infectivity under acidic bypass conditions. Cells were pretreated with either the solvent DMSO, posaconazole (Posa) or itraconazole (Itra) for 16 h, and were infected with IAV (PR8M, 20 MOI, 8 h) in the presence of Bafilomycin A1 under acidic bypass conditions. Non-infected cells and infection without acidic bypass in the presence of Bafilomycin A1 served as controls. NP-positive cells were detected by FACS (5,000 cells per sample). Data obtained in (A) A549 cells and (B) Vero cells are expressed as the median percentages ± SEM *n* = 5. ****, *p* ≤ 0.0001, two-way ANOVA with by Dunnett’s multiple comparison test.

To delineate which early step in the IAV uptake was impaired, we performed acidic bypass infection experiments. This technique forces the artificial fusion of the viral envelope with the host cell plasma membrane in an acidified environment. As a consequence, the viral genome is directly transferred into the host cell cytoplasm, thus evading endocytic uptake and virus/endosome fusion [34]. Simultaneous treatment with Bafilomycin A1 was used to inhibit IAV endocytosis. As shown in Figure 6, only neglectable levels of background staining were detected in the non-infected cells (see Suppl. Fig. 4 for gating strategy), and Bafilomycin A1 incubation potently inhibited IAV infection even under physiologic conditions. Under acidic bypass conditions, a robust number (23%) of DMSO-treated control cells were NP-positive. Interestingly, we detected a significantly lower number (∼15%) of infected A549 cells after pretreatment with both antifungal drugs. Notably, no impairment was observed in the interferon-insensitive Vero cells, suggesting that the activation of the interferon response by both drugs caused a compromised import of vRNPs into the host cell nucleus.

### Itraconazole improves survival upon IAV infection

**Figure 7.**
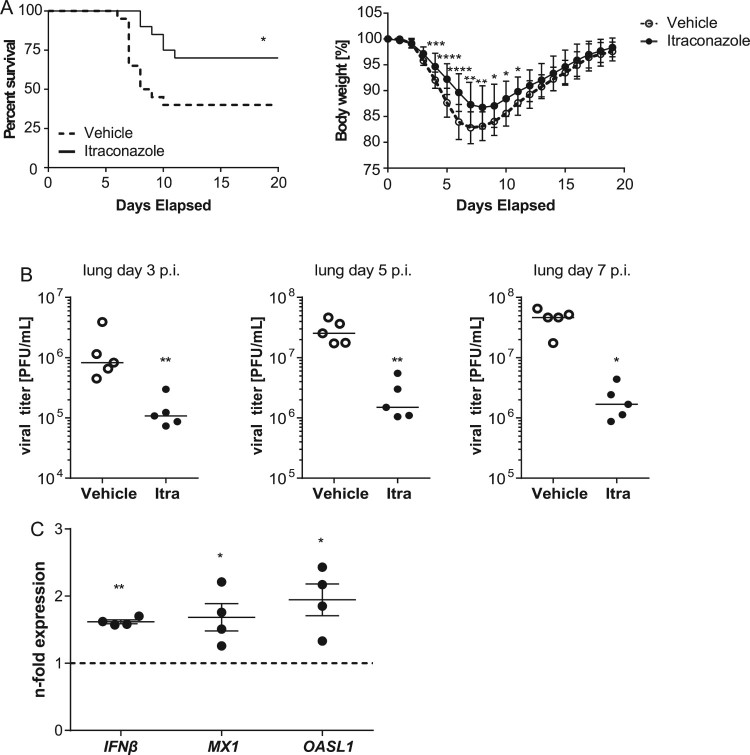
Itraconazole treatment restricts progression of IAV infection in mice. Eight-to-ten weeks-old male mice were treated intragastrically with itraconazole (70 mg/kg body weight) diluted in propylene glycol hydroxypropyl-β-cyclodextrine (Itra, solid line). Control animals received the equivalent volume of the solvent (vehicle, dotted line). Mice were infected intranasally with 500 pfu of the IAV H1N1 strain PR8M. (A) Cumulative survival rate and loss of liveweight plotted against days post infection. Mortality also includes mice that were euthanized because of a body weight loss of ≥20%. *n* = 20 mice/group, Mantel-Cox log rank test, *p* = 0.0136 or Mann–Whitney *U*-test. (B) Viral loads in lungs of the individual mice at indicated times post infection. (C) qPCR analysis of *IFNβ* and the ISGs *MX1*and *OASL1* in lung homogenates of non-infected mice treated with either vehicle (control) or itraconazole. Samples were obtained from four individuals per group and were run in triplicates. Expression levels of the genes of interest in the individual samples were normalized to GAPDH and CYCS. 2^−ΔΔCt^ was used to calculate the drug-induced fold change of relative gene expression compared to control animals. Graphs show difference in the respective genes in individual drug-treated animals relative to control, with the mean fold change ± SEM superimposed. Statistical significance of the differences was evaluated by unpaired student *t-test* on ΔΔCt values. *p < 0.1, **p < 0.01, ***p < 0.001, ****p < 0.0001.

Because itraconazole showed a more pronounced antiviral effect in the cell culture model, we focused on this drug for the transfer into the animal model. For extrapolating therapeutic doses among species, we used allometric scaling and the dose by factor method based on species conversion factors[35], resulting in a human equivalent dose (HED) of 5.7 mg/kg body weight, equivalent to 340 mg for a 60 kg human. Thus, mice that had received an intragastrical application of itraconazole (70 mg/kg body weight) or vehicle the day before were infected intranasally with LD_50_ of PR8M (500 pfu). Drug treatment continued for another 7 days p.i., and the course of infection was followed for a total of 20 days. In line with the inhibitory effects observed in the cell culture models, we observed a clearly reduced mortality in itraconazole-treated mice (70% survival) compared to the vehicle group (40% survival). The improved survival correlated with diminished body-weight loss during the course of IAV infection (Figure 7(A). To further examine the protective role of itraconazole in the host defense against IAV, we surveyed the viral burden in the respiratory tract. Accordingly, itraconazole-treated mice showed lower viral titers in lungs (Figure 7(B)) and tracheae (Suppl. Figure S5) at d3, d5, and d7 p.i., confirming that itraconazole could effectively control the spread of IAV infection. To explore the itraconazole-mediated effect on the IFN system in the lung, we performed qPCR analysis for *IFNβ* and the ISGs *MX1 and OASL1* on lung homogenates of drug-treated mice (Figure 7(C)). In line with our observations on a weak induction seen in the cell culture samples, we detected a moderate induction of the IFN response. These observations are a clear indication that the antiviral effects observed *in vitro* actually occur *in vivo*, and indicate a therapeutic potential of at least the antifungal drug itraconazole in the treatment of IAV infections.

## Discussion

IAV is a major threat to public health, mainly because of the annual epidemics, but also because of rare but severe pandemics [36,37]. The annual vaccine provides protection against currently circulating strains, but fails in a pandemic situation. Very few antiviral drugs are licensed and available for the treatment of influenza. Therefore, both the emergence of new strains and the acquisition of resistance poses problems which require novel solutions [2]. In this regard, the interactions of the virus with components of the host cell are increasingly being discussed as targets of new antiviral therapies [5]. Some of these host cell processes are targeted by existing drugs for other diseases [38]. Thus, drug repurposing, the re-use of known drugs in novel applications, can accelerate the development of new antiviral drugs against IAV [39,40]. In the present study, we investigated the use of the antifungal drugs itraconazole and posaconazole in the treatment of IAV infection. As expected with such established and widely used drugs, we did not detect any toxicity. Most importantly, both compounds were found to be highly effective in limiting IAV infection, with their EC_50_ values below the micromolar value.

Notably, changes in cellular cholesterol are linked with the immune system both as a consequence and as a cause [16], as a disrupted cholesterol biosynthesis triggers the activation of the IFN-mediated innate immune response [16,41]. Both types of medication interfere with the ergosterol synthesis pathway of the host cell [21,22]. Both compounds inhibit cytochrome P450 (CYP) enzymes, particularly the lanosterol 14α-demethylase, which is not only essential for the ergosterol biosynthesis in fungi but also impairs cholesterol homeostasis and *de-novo* synthesis in mammalian cells. A crucial role of cellular cholesterol in the defense against pathogens, and in particular against many viruses, has been shown in numerous studies [18,31,42,43]. Disturbed ergosterol metabolism shifts the tightly balanced type I IFN expression levels [44] toward induction of a pre-activated state, thereby accelerating the virus-induced host cell response. However, the unaltered cholesterol contents detected in itraconazole and posaconazole-treated cells suggest that the antiviral effect is not due to a disturbed cellular cholesterol biosynthesis. Of note, itraconazole was identified as a small molecule inhibitor of the endosomal cholesterol transporter NPC1 that directly binds to the sterol-sensing domain of NPC1, resulting in cholesterol accumulation in LE/L [18]. To release the viral genome into the cytosol of the host cell, the lipid envelope of IAV fuses with the membrane of the acidified late endosomes [45–47]. Exactly at this cellular site, which is critical for the IAV infection, a drug-induced high accumulation of cholesterol was observed, consistent with previous publications [19,48]. The significance of elevated LE/L cholesterol levels for IAV endosomal escape is still controversially discussed [18,49]. However, there is strong evidence that elevated levels in this compartment negatively impact viral genome transfer into the host cell cytosol [24]. Additionally, shifting the cholesterol homeostasis towards the LE/L compartments diminishes the levels of cholesterol in the host cell plasma membrane, causing decreased cholesterol contents in the envelope of the nascent virus progeny, which reduces infectivity of newly released IAV particles [10,50]. A solid body of evidence has already shown that the balance of cellular cholesterol is a key determinant in the infection of a wide range of viruses [10–14]. In this regard, itraconazole was shown to function as a broad-spectrum inhibitor for enteroviruses through modulation of the host cholesterol homeostasis [51]. Our data contradict a study by Shim et al. [52]. Contrary to our experimental set-up, which measures the inhibitory effect on the exposure to a defined viral load by determining the amount of virus released after 24 h by a plaque assay, in this study an assay based on the reduction of a cytopathic effect (CPE) was used to determine potential inhibitory effects after 2 days of incubation with the virus. It is difficult to evaluate from these data whether the correlation of cell death with virus titers was still in a dynamic range, and an inhibitory effect on viral replication might have remained undetected. In line with this assumption, we also observed a more obvious inhibitory effect at the lower viral doses (0.005 MOI). Furthermore, after 3 additional rounds of replication, even reduced numbers of viral progeny might have been still sufficient to kill nearly the whole population (because viral replication is exponential).

Remarkably, endolysosomal cholesterol accumulation as an early antiviral barrier seems to be embedded in the antiviral response initiated upon type I IFN exposure [24]. The cholesterol imbalance is most likely why drug treatment moderately but significantly increased the expression of both IFN itself and IFN-stimulated genes in non-infected cells. In the context of infection, large amounts of type I IFNs are rapidly, yet transiently, produced. However, little is known about the host cell-mediated induction of IFNs via endogenous inflammatory factors in the absence of pathogens. Notably, low but sustained IFN production (picomolar concentrations) is already sufficient to induce a priming and thus an enhanced immune response upon microbial challenge, at least in macrophages [53]. Such a host-elicited priming mechanism might be of special importance during the early infection phase, when IFN levels are still low, thus allowing primed cells to mount the antiviral response more rapidly. Interestingly, our data on the upregulation of IFN and select ISGs in drug-treated and subsequently IAV-infected cells compared to vehicle-treated infected cells suggest a priming function of the drugs, which might contribute to the observed inhibitory effect of itraconazole on IAV infection. However, we cannot exclude that the priming is elicited via drug-induced upregulation of other endogenous inflammatory factors that cause a subsequent IFN expression. Of note, the weak induction of *IFNβ* and the ISGs was also detected in lungs of itraconazole-treated animals, indicating that itraconazole-mediated priming occurs *in vivo*.

IFN treatment causes a general antiviral state and confers protection against a broad range of unrelated DNA and RNA viruses [54,55], yet the IFN-induced gene products are often virus-selective [56]. Mechanistically, the specificity of the antiviral effect might depend on the IFN signature. Interestingly, itraconazole, which had a more pronounced antiviral effect, elicited *IFNβ* upregulation more efficiently than posaconazole. However, the comparison of the data obtained in A549 cells and IFN-insensitive Vero cells clearly demonstrates an IFN-independent inhibitory mode of action. Appropriately, an inhibitory effect of drug pretreatment on IAV infection bypassing the endosomal route occurred only in A549 cells capable of IFN response and not in Vero cells. Thus, we assume that the potent inhibition of IAV infection in the first replication cycle in A549 cells presumably results from both components, the impaired endosomal escape and a weak priming of the IFN response. Importantly, itraconazole reduced IAV infection *in vivo* in the mouse model. The estimated HED was equivalent to 340 mg for a 60 kg human. As the recommended daily dose of itraconazole for therapy of fungal infections is 200–400 mg/day, and might be increased up to 600 mg/day for adults and severe infections, the dose used in the animal infection model is well within the range used in the treatment of fungal infections, and less than the maximum dose in human.

Because viruses often encode only very few proteins of their own, they need to efficiently hijack the host cell machinery. The notion that the drug elicits antiviral activity by targeting more than one host cell mechanism may be advantageous, because this will also target more than one step in the viral life cycle. Moreover, such an approach will most likely cover a broader spectrum of strains which differ in their extent to which they depend on the cellular drug targets, and also circumvent viral adaptations to the IFN-induced antiviral state. Our study, which not only reports an inhibitory effect of both itraconazole and posaconazole in cell culture but shows an efficient itraconazole-mediated reduction in IAV infection *in vivo* in the mouse model at a HED used in the treatment of fungal infections in humans, may offer a promising application potential towards the treatment of IAV infections.

## Materials and methods

### Cells and drug treatment

The human type II alveolar epithelial cell line A549, the human epithelial carcinoma cell line A431, the interferon-deficient Vero cells and the Madin-Darby canine kidney (MDCK) cells type II were cultivated in Dulbecco’s modified Eagle’s medium (DMEM) supplemented with 10% standardized fetal bovine serum (FBS Superior; Merck Cat. No. S0615), 2 mM L-glutamine, 100 U/ml penicillin, and 0.1 mg/ml streptomycin. All cell lines were cultured in a humidified incubator at 37°C and 5% CO_2_. Itraconazole (Sigma) and posaconazole (Sigma) were solubilized in DMSO at 2 mg/ml. Prior to infection, cells were treated with 2 µg/ml itraconazole or posaconazole for 16 h, or with 250 nM Bafilomycin A1 (Cayman-Cay-11038) for 2 h. For postinfection treatment, 2 µg/ml itraconazole or posaconazole were added 2 h postinfection.

### Cytotoxicity assay

A549 cells were untreated or treated at the indicated concentrations with the solvent DMSO, itraconazole, and posaconazole. Because of the strong cytotoxic effects, staurosporine (1 µM) served as a positive control. Upon 24 h of treatment, MTT 3-(4,5-dimethylthiazol-2-yl)-2,5-diphenyltetrazolium bromide was added to the cells for 4 h. The supernatant was aspirated and DMSO was added for 5 min, and subsequently, the OD_562_ was measured.

### Viruses and infections

The human influenza virus strains A/Puerto-Rico/8/34 Münster (H1N1) (PR8M), seasonal A/Pan/99 (H3N2) (PAN), A/seal/Mass/1-SC35M/1980 (H7N7) (SC35M), B/Lee/1940 (Lee), and the vesicular stomatitis virus (VSV) strain were from the virus strain collection of the Institute of Virology, University of Münster, Germany. All virus strains were passaged and titered on MDCK cells. For infection, cells were washed with PBS and were infected at the indicated multiplicities of infection (MOI) of virus diluted in infection-PBS (PBS containing 0.2% bovine serum albumin (BSA), 1 mM MgCl_2_, 0.9 mM CaCl_2_, 100 U/ml penicillin and 0.1 mg/ml streptomycin) at 37°C (33°C for IBV) for 30 min. Cells were then washed with PBS and incubated with infection-DMEM (DMEM containing 0.2% BSA, 1 mM MgCl_2_, 0.9 mM CaCl_2_, 100 U/ml penicillin, and 0.1 mg/ml streptomycin) for the indicated periods of time.

### Plaque titration

Tissue from infected mice was harvested at various times post infection (3, 5, or 7 days p.i.) and homogenized utilizing Lysing Matrix (MD Biomedicals) according to the manufacturer’s protocol. Numbers of infectious particles in the supernatants of tissue homogenates or infected cells were determined via plaque assay technique.

### Single-cycle infection analysis (NP assay)

A549 cells were infected with PR8M, and fixed with 4% paraformaldehyde (PFA) in PBS for 10 min at room temperature at the indicated times post infection. To visualize the viral nucleoprotein (NP), cells were permeabilized with 0.1% TritonX-100 in PBS for 30 min, blocked with 2% BSA in PBS for >1 h, and stained with FITC-coupled anti-NP antibody (clone Sigma Aldrich, 1:50 in 2% BSA in PBS) for 2 h. Cell nuclei were stained with DAPI (Thermo Scientific, 1:1000 in 2% BSA in PBS) for 10 min. Cells were analysed by confocal microscopy using an LSM 800 microscope (Carl Zeiss, Jena, Germany), equipped with a Plan-Apochromat 63x/1.4 oil immersion objective. Quantitative analysis was performed on the images utilizing CellProfiler, an open-source cell analysis tool for quantitative measurement of cell phenotypes [57].

### Acidic bypass

The applied assay was established by Stauffer et al. (2014). Prior to infection, cells were pre-incubated with Bafilomycin A1 (Cayman, Cay-11038, 250 nM). PR8M virus was primed for 1 h at 37°C with low pH-DMEM (without supplements) adjusted to pH 5.8 with MES (30 mM). Cells were infected with 20 MOI of the primed virus for 1 h on ice while shaking moderately, and subsequently washed twice with cold infection-DMEM (DMEM containing 0.2% BSA, 1 mM MgCl_2_, 0.9 mM CaCl_2_, 100 U/ml penicillin, and 0.1 mg/ml streptomycin). Acidic bypass was achieved by incubation with prewarmed low pH-DMEM (50 mM citrate, pH 5.0) for 2 min at 37°C, followed by two washing steps with cold infection-DMEM. Infection-DMEM containing Bafilomycin A1 (250 nM) and the tested substances was added and cells were incubated for 8 h at 37°C. For FACS analysis, the cells were harvested by Accutase treatment (Millipore, SCR005) and transferred to 96-well plates with V-bottom (Sigma, CLS3896). After centrifugation at 300 rcf for 4 min at 4°C, cells were fixed in 4% PFA for 10 min at RT and permeabilized by 0.1% Triton X-100 for 10 min at 4°C. Blocking was performed with 2% BSA overnight at 4°C. Cells were stained with anti-NP-FITC (clone Sigma Aldrich, 1:50 in 2% BSA in PBS^+/+^) for 2.5 h. Cells were washed with PBS^+/+^ three times. For fluorescence cytometry analysis, 5000 cells per sample were inspected. Uninfected cells or cells without acidic bypass served as control.

### Filipin staining and colocalization analysis

Cells were fixed with 4% PFA in PBS at room temperature for 10 min, subsequently washed twice with PBS, and blocked with 2% BSA in PBS for 60 min. To visualize free cellular cholesterol, cells were stained with filipin (complex from *Streptomyces filipinensis*, 1.25 mg/ml) for 2 h. Late endosomal/lysosomal compartments were identified by staining the endolysosomal marker protein CD63 with a TRITC-coupled anti-CD63 antibody (Santa Cruz Biotech; 1:50 in 2% BSA in PBS) for 90 min. Cell nuclei were labelled with DRAQ5 (1:1000 in 2% BSA in PBS) for 30 min. Confocal microscopy was performed with an LSM 780 microscope (Carl Zeiss, Jena, Germany) equipped with a Plan-Apochromat 63x/1.4 oil immersion objective. The colocalization of filipin and CD63-TRITC signals were quantified with the JACoP plugin [58] for Fiji [59].

### Quantification of cellular cholesterol contents

Cellular cholesterol contents were quantified using the Amplex Red cholesterol assay kit (Invitrogen) as described previously [14,24].

### Quantitative reverse transcription PCR

For cell culture experiments, total cellular RNA was isolated from seven independent samples per condition using the RNeasy Kit (Qiagen) according to the manufacturer’s instructions. cDNA was synthesized with the RevertAid H Minus First Strand cDNA Synthesis Kit (Thermo Fisher Scientific) according to the manufacturer’s protocol. Quantitative reverse transcription PCR (RT-qPCR) was performed using the Brilliant III Ultra-Fast SYBR Green QPCR Master Mix (Agilent Technologies) and a Roche LightCycler 480 according to manufacturer’s instructions. The applied primers were the following: GAPDH_fw: 5′-GCAAATTCCATGGCACCGT-3′, GAPDH_rv: 5′-GCCCCACTTGATTTGGAGG-3′, Hs_ACTB_1_SG QuantiTect Primer QIAGEN (Cat. No. QT00095431), IFNα_fw: 5′-ATACCTTTGCCACCCGAAAACT-3′, IFNα_rv: 5′-CTTCAAAGCC AGTGGCACTCAT-3′, IFNβ_fw: 5′-GGCCATGACCAACAAGTGTCTCCTCC-3′, IFNβ_rv: 5′-GCGCTCAGTTT CGGAGGTAACCTGT-3′, MxA_fw: 5′-GTTTCCGAAGTGGACATCGCA-3′, MxA_rv: 5′-GAAGGGCAAC TCCTGACAGT-3′, IFITM3_fw: 5′-ATGTCGTCTGGTCCCTGTTC-3′, IFITM3_rv: 5′-GTCATGAGGATGCCCAGAAT-3′.

Lungs of four animals per condition were homogenized in TriZol using lysing matrix tubes (MD Biomedicals). Total RNA was isolated and 1 µg was converted into cDNA using the High-capacity cDNA reverse transcription kit and random hexamer primers (Applied Biosystems). Antiviral gene expression in the lung was analysed by murine Taqman primer/probe sets (Universal ProbeLibrary, Roche) on a LightCycler^®^ 480 Instrument II (Roche).

For all qPCR setups, samples from independent experiments were run in triplicates. For statistical analysis, the delta-delta-Ct method was used. In brief, Ct values for genes of interest in the individual samples were normalized to the housekeeping *genes GAPDH (Glyceraldehyde-3-Phosphate Dehydrogenase)* and *ACTB (beta-Actin)* or, in the case of lung homogenates, *GAPDH* and *CYCS* (*Cytochrome c*), resulting in ΔCt values. Mean ΔCt of the control samples was calculated, and ΔΔCt values of all individual samples were calculated as difference between the ΔCt value of the individual sample and the mean control ΔCt. 2^-ΔΔCt^ was used to calculate the relative fold gene expression levels. One-way ANOVA on ΔΔCt values was used to analyse the statistical significance of the differences.

### *In vivo* IAV infection of mice

WT C57BL/6 mice were purchased from Envigo (The Netherlands) and housed at the barrier-free and specific pathogen-free facility at the Center for Molecular Biology of Inflammation (ZMBE) at the University of Muenster. All animal experiments were performed in compliance with the guidelines for the welfare of experimental animals issued by the Federal Government of Germany and the state of North Rhine-Westphalia and were approved by an external committee (LANUV, 84-02.04.2016.A426). Eight-to-ten weeks-old male animals were treated intragastrically with itraconazole (70 mg/kg body weight) diluted in propylene glycol hydroxypropyl-β-cyclodextrine. Human equivalent dose (HED) was calculated using the equation HED mg/kg = mouse dose mg/kg × *K*_m_ ratio, with a *K*_m_ for mouse/man conversion of 0.081[35]. Control animals received the equivalent volume of the solvent. Mice were anesthetized with isoflurane prior to intranasal infection with 500 pfu (plaque forming units) of the IAV strain PR8M. To assess pain and distress during the course of infection, animals were assessed based on a scoring system with sufficiently frequent observation times that assigns numerical values to several criteria of animal conditions that were considered signs of morbidity or moribundity, including changes in body temperature, physical appearance, behaviour, and weight loss. Animals that reached the cumulative threshold score were euthanized. A body weight loss of >20 percent compared to start of the treatment was the cut-off parameter for euthanasia, regardless of the total score. From a total of 40 infected mice (20 itraconazole-treated, 20 vehicle-treated), 18 mice had to be euthanized because body weight loss was more than 20%. The date of death for euthanized mice was marked as the date of euthanasia. None of the animals was found dead during the course of the experiment.

### Data analysis

A priori power analysis (G*Power 3.1, Faul et al., 2007) was used to estimate the required sample sizes. Data were analysed with Prism 6.00 (GraphPad). For dose–response curves, virus titers were normalized to percentages of the titers detected in control cells, and drug concentrations were log-transformed. IC values were calculated from the sigmoidal curve fits. For statistical analysis, significant differences were evaluated using one-way ANOVA followed by Dunnett’s multiple comparison test. *In-vivo* experiments were analysed by Mantel-Cox log-rank test or Mann–Whitney *U*-test. *p < 0.05, **p < 0.01, ***p < 0.001.
